# The Critical Need for a Meaning-Centered Team-Level Intervention to Address Healthcare Provider Distress Now

**DOI:** 10.3390/ijerph19137801

**Published:** 2022-06-25

**Authors:** William E. Rosa, Kailey E. Roberts, Amelia E. Schlak, Allison J. Applebaum, William S. Breitbart, Emily H. Kantoff, Hayley Pessin, Wendy G. Lichtenthal

**Affiliations:** 1 Department of Psychiatry and Behavioral Sciences, Memorial Sloan Kettering Cancer Center, 641 Lexington Avenue, 7th Floor, New York, NY 10022, USA; applebaa@mskcc.org (A.J.A.); breitbaw@mskcc.org (W.S.B.); kantoffe@mskcc.org (E.H.K.); pessinh@mskcc.org (H.P.); lichtenw@mskcc.org (W.G.L.); 2 Ferkauf Graduate School of Psychology, Yeshiva University, 1165 Morris Park Avenue, Bronx, New York, NY 10461, USA; kailey.roberts@yu.edu; 3 School of Nursing, Columbia University, 560 West 168th Street, New York, NY 10032, USA; aes2355@cumc.columbia.edu

**Keywords:** burnout, moral distress, meaning-centered psychotherapy, resilience, COVID-19

## Abstract

COVID-19 has unveiled and amplified the burnout, grief, and other forms of distress among healthcare providers (HCPs) that long preceded the pandemic. The suffering of the healthcare workforce cannot be simply and sufficiently addressed with a single psychotherapeutic intervention. Nevertheless, the National Academies of Sciences, Engineering, and Medicine Studies recommended prioritizing interventions that generate an increased sense of meaning in life and in work to reduce burnout and cultivate clinician wellbeing. Despite their guidance, there is a dearth of interventions for HCPs specifically targeting meaning and purpose as an avenue to reduce HCP distress. In a time when such an intervention has never been more essential, Meaning-Centered Pyschotherapy (MCP), a brief, evidence-based intervention designed for patients with advanced cancer may be key. This piece describes the principles underlying MCP and how it might be adapted and applied to ameliorate burnout among HCPs while providing a rationale to support future empirical studies in this area. Importantly, the systemic factors that contribute to the emotional and mental health burdens of HCPs are discussed, emphasizing the need for systems-level changes that are needed to leverage the potential outcomes of MCP for HCPs.

## 1. Introduction

Healthcare providers (HCPs) are drowning. The World Health Organization (WHO) recognizes that HCPs’ “levels of anxiety, distress, fatigue, occupational burnout, stigmatization, exposure to physical and psychological violence from communities have all increased significantly” in the wake of the COVID-19 pandemic [1]. They estimate that roughly 115,500 HCPs died as a result of the pandemic between January 2020 and May 2021 [1], a number that does not account for deaths secondary to individual and occupational stressors, including suicide, or long-term sequelae of infection. These deaths are–in part–a result of the inability or unwillingness of international government and healthcare leadership to prioritize the health and safety of HCPs at local and systems levels. This moral failing to strategically and sustainably invest in the wellbeing of HCPs adds further strain to an already precarious and maxed-out system characterized by insufficient fiscal and human resources.

Beyond the fears and risks associated with contracting SARS-CoV-2, HCPs across disciplines are confronting work-related and personal distress, including moral injury and burnout, at unprecedented levels [2,3]. As they witness profound suffering in their patients, HCPs in turn suffer—confronting chronic threats to their own health and laboring in overwhelmed hospital, community-based, and long-term care settings. Although the urgency of some COVID-19 related concerns may shift over time, with less threatening variants and improved public health policies, others–such as significant proportions of some populations refusal to be vaccinated–will likely continue to have negative impacts.

Several forms of HCP work-related distress, including moral suffering and secondary traumatic stress, have been well-documented for decades and are due to a variety of circumstances, including understaffing, poor work environments [4,5,6], and the witnessing of patient suffering and death [7]. One type of distress that has continued to garner particular attention in the wake of the pandemic is burnout, described as a multifactorial problem requiring a multi-pronged approach [8]. The rates of clinician burnout were strikingly high prior to COVID-19, with rates up to 40% [9] in physician and 35–40% in nurses [10,11,12] and affected frontline providers [13], as well as trainees and students [14]. Furthermore, the consequences of HCP distress extend beyond the individual. For example, nurse burnout has been linked with higher rates of patient mortality [15] and hospital acquired infection [16] and, at the organizational level, with increased turnover [17], costing organizations millions of dollars each year [18].

These psychosocial, emotional, and system-level burdens have only become more pervasive in the face of the COVID-19 humanitarian crisis [19,20]. Findings from a recent survey of more than 6000 acute and critical nurses conducted by the American Association of Critical-Care Nurses carry dire implications for both HCP health and workforce sustainment: 67% of nurses expressed, “I fear taking care of patients with COVID puts my family’s health at risk;” 76% noted, “People who hold out on getting vaccinated undermine nurses’ physical and mental well-being;” and 92% described, “I believe the pandemic has depleted nurses at my hospital. Their careers will be shorter than they intended” [21]. The pandemic has amplified the already-critical issue of burnout in HCPs, leaving all too many disillusioned and disconnected from the sense of meaning and purpose they typically derive from their work. The aim of this essay is to describe the principles underlying Meaning-Centered Psychotherapy (MCP)–a brief, evidence-based intervention designed for patients with advanced cancer–and how it might be adapted and applied to ameliorate burnout among HCPs while providing a rationale to support future empirical studies in this area.

## 2. Meaning and Purpose: Rediscovering Our ‘Whys’

A higher calling and sense of duty are strong contributing factors to why many HCPs pursue a career focused in healthcare in the first place [22,23]. When HCPs’ sense of meaning is challenged, their “whys” for doing this fulfilling, yet hard, work places our healthcare system at risk of losing its very best [24]. Studies have shown that greater meaning in life (e.g., meaning salience) among multidisciplinary HCPs is associated with less fatigue and burnout and a lower burden of psychological distress, as well as a greater positive affect and improved quality of life [25,26]. Thus, the need for supportive interventions that directly target meaning and purpose to reduce HCP distress across health disciplines has never been so critical. In fact, the National Academies of Sciences, Engineering, and Medicine identify meaning and purpose in work as one of the key work system factors that predict burnout. In their 2019 report, *Taking Action Against Clinician Burnout,* they call for health systems to adopt innovations that “enhance meaning and purpose” for HCPs in their work as a means to promote wellbeing and patient care quality (Recommendation 1B) [8].

Despite this call to action, and the fact that maintaining a sense of meaning has been shown to buffer against stress in HCPs [25,26], there are few interventions targeting the enhancement of meaning as an avenue for improving HCPs’ wellbeing [27,28]. A recent systematic review found existing interventions that have demonstrated efficacy in targeting burnout have largely focused on communication skills and basic stress management [28]. Additionally, studies on HCP interventions have shown that enhancing collegial relationships is critical [29]. A team-level, meaning-based intervention has the potential to go beyond the promotion of communication and coping skills to support HCPs in deeply connecting around a shared sense of meaning, which may in turn improve team-level functioning through uniting around what is most important to individual HCPs and the team [30]. It is time to address this gap through evidence-based approaches to alleviate distress for these highly burdened, multidisciplinary populations.

## 3. Meaning-Centered Psychotherapy for Healthcare Providers

Our team at Memorial Sloan Kettering Cancer Center (MSK) is currently taking steps to adapt Meaning-Centered Psychotherapy (MCP) for HCPs (MCP-HCP) to mitigate HCP distress such as burnout and bolster wellbeing. MCP is a manualized, brief psychotherapeutic intervention, originally developed by Breitbart and colleagues [31,32,33,34,35] to enhance a sense of meaning and purpose in patients with advanced cancer and help them face adversity, ultimately improving their quality of life. The principles of MCP are grounded in work by existential psychiatrist, Viktor Frankl [36], who defined meaning as the conviction that one can create a life with meaning and purpose through connectedness with valued relationships, roles, activities, and experiences in the world. Specifically, individuals are encouraged to explore what matters most to them and to find ways to connect to sources of meaning, including connecting to others, in spite of the challenges they may be facing. They are supported in addressing barriers to connecting whenever relevant.

Through didactics about sources of meaning, or lenses from which to understand meaning in life, and experiential exercises to help integrate these ideas into daily life, MCP helps individuals to connect with what is most important to them and to explore how their sources of meaning may buoy them through adversity. MCP begins with an exploration of meaningful moments in an individual’s life and of their authentic sense of identity. With this foundation, MCP then takes individuals through discussion of their sources of meaning. The first source of meaning is the impact of one’s history on their authentic sense of self and the legacy they put into the world. MCP then engages individuals in highlighting the ways in which. when circumstances such as systems-level workplace challenges, are beyond one’s control, they can focus on what they do have control over and how they choose to respond to the adversity they face. It applies existential principles of free will and taking care of their existence, placing them in an empowered position to respond effectively to difficult circumstances. Shifting from the meaning that can be found in the perspective one takes, MCP then empowers individuals to honor the ways in which they have demonstrated courage in committing to creative a life of meaning, whatever that looks like for them. This concept of “creative sources of meaning” may be particularly powerful for HCPs who, when in the thick of the numerous challenges of just getting through each day, may minimize or not have the time to acknowledge their accomplishments and contributions. Finally, MCP concludes with an exploration of experience as a source of meaning or the idea that by noticing the small moments of beauty, love, and humor, it is possible to connect to meaning, even if momentary. These small moments can be a reminder in the moment that life has meaning and can multiply to bring a sustained sense of meaning to suffering HCPs. 

When delivered in a group format where members have shared experiences or are confronting similar challenges, MCP offers not only the opportunity to describe these individual sources of meaning, but also to enhance connectedness and perhaps even their feeling of belonging to something greater than oneself. MCP group members often recognize sources of meaning in themselves when hearing the stories and responses of other members. This reciprocal bolstering, expanding, and deepening of the members’ sense of meaning and connectedness can also bring a sense of shared meaning and purpose to the group more broadly.

MCP can be distinguished from other work-related interventions based on cognitive, behavioral, and/or positive psychology and resilience principles because of its existential lens. Through structured reflection questions, MCP guides individuals in discussions about meaning in life, one’s unique sense of identity and purpose, how to apply the concept of “free will” and choice in one’s work and personal life, and, importantly, how meaning can sustain us through challenging times. It can help HCPs tap into the “why” of it all. A key component to effectively delivering MCP is to avoid being overly optimistic or minimize harsh realities, critiques which have been suggested about resilience work [37] and to keep the focus on what is authentically meaningful to a given person in the context of their particular challenges. Relatedly, it is also not necessarily a positive psychology intervention as the MCP approach acknowledges that meaning is not always inherently positive. Rather, meaning is what is most important to us, the reasons we continue on despite the challenges we may endure. HCPs, for example, could find their work meaningful but not experience it as something that imbues them with happiness on a daily basis. This existential framework, paired with a highly structured format seen in many evidence-based psychological interventions, is predominantly unique to MCP [38] and has not been a feature of existing interventions targeting burnout [28].

In addition to being a distinct approach, MCP has a strong evidence base. Through randomized controlled efficacy trials comparing MCP to supportive psychotherapy [35,39], both individual and group formats of MCP have demonstrated efficacy in increasing spiritual well-being and quality of life and improving psychosocial outcomes among patients with advanced cancer, with the enhancement of meaning being the mediating factor in reducing anxiety, depression, and the desire for hastened death [31,34,35,39]. Given this strong evidence-base, MCP has been designated by the National Cancer Institute (NCI) as an Evidence-Based Cancer Control Program and numerous adaptations have been developed for other populations without active medical illness, including for breast cancer survivors, cancer caregivers, and bereaved individuals [40,41,42,43]. MCP is, thus, poised to be a promising intervention for HCPs, particularly in supporting HCPs to maintain meaning when navigating the unavoidable sources of distress they experience as part of their clinical work (i.e., witnessing patient suffering and death) and for improving team connectedness through shared reflection [44].

Importantly, we acknowledge that the existential distress resulting from facing impending death for oneself or a loved one for which MCP was originally developed is not the same as facing untenable working conditions (which must ultimately be rectified at the systems-level). Research has shown that making change solely at the intrapersonal level is not an effective, long-term strategy for targeting burnout because the systems-level factors contributing to burnout are too powerful for a single individual to overcome [45,46,47,48,49]. Consensus from policy experts and researchers suggests that the systems-level factors contributing to burnout and clinician distress are avoidable if targeted and addressed by health systems leaders; however, systems-level change is often slow and requires stakeholder buy-in. In the meantime, the downstream effects are being experienced by individual HCPs and causing significant distress.

Thus, in order to be optimally effective and disseminated, MCP-HCP will need to factor in healthcare system-level concerns. As an intra- and inter-personal intervention, MCP-HCP cannot change the system-level context to prevent upstream causes of burnout (i.e., poor working conditions), but MCP-HCP offers an opportunity to center the growth of HCP teams’ sense of connection and agency to empower and better equip them for these systemic challenges. While MCP for individual patients emphasizes drawing on sources of meaning to cope with the challenges of life-limiting illness, MCP-HCP aims to take a team-oriented perspective and draw on the power of meaning-centered principles to improve team functioning and collegiality through shared reflection and empowerment. Facing existential distress, grief, and the stressors inherent to healthcare work alone may be isolating or overwhelming; but connecting on the most human and deepest of levels in a group context and reflecting on challenges and meaning collectively may promote empathy, camaraderie, transparency, community-building, and both individual and team agency. Fostering a sense of connectedness and the opportunity to reflect as a group about common challenges HCPs face in their work as well as points of existential distress and sources of meaning that could buoy them through these challenges among HCPs may, in turn, promote the healing environment for patients [6,50]. Facilitating such collegial connection through meaning is in line with research that demonstrates a sense of connection among colleagues is a critical component of a positive working environment and, in turn, a healing environment for patients [6,50]. With its focus on connectedness as a key source of meaning and strong evidence-base for group-delivered MCP, it may thus hold promise to support HCP teamwork.

The potential power of MCP to facilitate team connectedness and meaning-making was witnessed during informal meaning-focused groups we offered in our institution at the height of the COVID-19 pandemic in New York City; clinical staff articulated numerous issues contributing to exacerbated stress and burnout. Themes that emerged included anxiety and helplessness regarding SARS-CoV-2 transmission risk; experiences of grief and loss; conflicting emotions of self-preservation vs. delivery of compassionate high-quality care; feelings of stress related to resource constraints and inadequate support; “health hero” labeling in the absence of alternative work options; sentiments of fear, isolation, loneliness, anger, and uncertainty; existential guilt or “wishing I could do more”; and anxiety related to rapid changes in infection rates and related policy actions. While engaging in MCP together did not take away these profound challenges, the sharing of mutual points of existential distress appeared to foster a connectedness between the HCPs and an opening to come together to maintain a sense of meaning in the face of suffering.

Direct and sustained collaboration with HCPs will be crucial to guiding and maximizing the feasibility and efficacy of a meaning-centered intervention. Though a preliminary study applying MCP to palliative care workers was promising in their findings that MCP contributed to the HCPs experiencing a sense of enhanced meaning in work [51]; few further efforts been made to systematically adapt MCP for HCPs using rigorous intervention adaptation methods such as obtaining stakeholder and implementation feedback to ensure acceptability and uptake. For the past several years, our interdisciplinary team has been running an NCI R25-funded training program at MSK to equip clinicians in delivering MCP for patients. We have received numerous requests over the years for an adaptation of MCP geared toward HCPs. COVID-19 has increased the frequency and urgency of these requests. Clinicians trained in our R25 program have attempted to adapt MCP to support healthcare workers in their respective settings and have anecdotally reported that attendees experienced decreases in distress and an increased sense of hope. While these informal efforts have been promising, clinicians have consistently expressed a desire for a formally tailored approach that is empirically validated. Therefore, we are employing rigorous mixed methods utilizing stakeholder feedback to tailor both the content of MCP-HCP and its delivery format to maximize its relevance, acceptability, and likelihood of uptake. We are also exploring diverse platforms to flexibly deliver the MCP-HCP content at HCPs’ convenience to increase feasibility and potential for dissemination and implementation.

## 4. Discussion and Future Directions: Meaning Is Necessary, but Not Sufficient

We acknowledge that “resilience” and “self-care” have become triggering concepts during the pandemic, given that HCPs have often reported feeling abandoned by multi-sectoral leaders and often have very little time or energy to devote to wellness [37,52]. Our team agrees that the liability of resilience capacity building should not be shirked and misdirected to the very individuals who are suffering, but rather promoted, procured, and advocated for by health systems leadership and policy makers. We do not intend to place yet another task on the already overburdened HCPs by implying the solution to this multilevel problem is for HCPs to make time to fix their burnout. Rather, we intend to work within the existing limitations and challenges that HCPs face to tailor a team-level intervention that acknowledges their humanity, their struggles, their strengths, their purpose, their ‘whys’, brings enhanced meaning and purpose amid system-wide suffering, and fosters choice within the constraints they face together–all of which we believe will alleviate suffering and existential isolation and promote team empowerment, efficacy, and advocacy.

The intention of the MCP-HCP adaptation is not to help clinicians tolerate toxic working conditions, but rather to provide them with a framework that fosters choice within a constrained and flawed system, as well as meaningful connections with each other. We intend on learning from stakeholders the optimal way of delivering MCP-HCP, respecting that there is likely variability in what is feasible and preferred across individuals and settings. While we have argued for the potential benefits of delivering MCP-HCP at a team level, we also recognize and have experienced the challenges inherent in many settings to bringing together HCP teams to engage in such an intervention. Thus, a critical component of future work on MCP-HCP must be to obtain and leverage HCP-, team-, and institutional-level feedback on effectively and sustainably implementing MCP-HCP.

It is also essential to recognize that individual HCPs will undoubtedly have vastly diverse health needs and experience a broad range of social health determinants that directly inform their well-being. While MCP-HCP is not a failsafe, our previous work [31,34,35,39,40,41,42,43,51,53,54], along with other research on meaning salience and HCP outcomes [25,26,55], strongly suggest that the ability to capitalize on choice and meaning-making, whatever that is uniquely to them, may help a person face adversity. As HCPs fulfill their professional responsibilities in the context of different intersectional identities and stressors, MCP-HCP may assist to bridge the gap between the realities of systemic constraint and the possibilities of personal and team agency.

Leveraging MCP to enhance meaning in HCPs is one aspect of the larger puzzle, intended to support clinicians in the challenges they face, but should not be considered a “free pass” to organizations seeking to avoid larger system-level changes. While meaning-making is an important way to help HCPs nurture their agency and choice, it is not a substitute for the larger policy and practice shifts changes that are essential for sustained HCP wellbeing. We hope that leadership can create space and carve out time for this so that engagement in the intervention is not yet one more thing on HCPs’ plates. As we continue to develop and disseminate MCP-HCP, it is ethically incumbent upon health systems to provide investments in psychosocial support for HCPs and, specifically, incentives for HCPs to use these services during working hours, and to strive toward a work culture that increases workforce discourse on the meaning behind healing and healthcare. The decisions of federal, state, and institutional policy makers have downstream effects on HCPs and thus, many health system burdens faced by HCPs are preventable at the system level if different investments are made [20].

## 5. Conclusions

We–as a healthcare community–cannot afford to not intervene with meaning-centered approaches to support our colleagues *now*. There is a tangible cost of not engaging healthcare workers in this way. We risk losing the most qualified, engaged, and compassionate staff both because of how much they give and their increased likelihood to self-sacrifice. As SARS-CoV-2 variants develop and severe cases continue, we need emotionally healthy staff to meet patient and family needs at the intersection of the pandemic and along the highly complex continuum of care. A stakeholder-informed MCP-HCP intervention has the potential to sustain dedicated frontline workers who are deserving of resources and science that truly “has their back.” This particular approach may foster a sense of hope and provide an empathic, human-centered response to supporting HCP teams in their commitment to serving society. In essence, we aim to deploy this intervention to gather needed empirical data while transforming our collective tragedy into a triumph and this predicament into an achievement of the human spirit.

## Data Availability

Not applicable.
